# Initiatives to promote access to medicines after publication of the Brazilian Policy on the Comprehensive Care of People with Rare Diseases

**DOI:** 10.1186/s13023-023-02881-5

**Published:** 2023-08-31

**Authors:** Cássia Cunico, Geison Vicente, Silvana Nair Leite

**Affiliations:** 1 Graduate Program in Pharmaceutical Policy and Services PPGASFAR, Universidade Federal de Santa Catarina – Campus Trindade, Florianópolis, Santa Catarina CEP 88.040-000 Brazil; 2 Graduate Program in Pharmacy, Universidade Federal de Santa Catarina – Campus Trindade, Florianópolis, Santa Catarina CEP 88.040-000 Brazil; 3 Graduate Program in Pharmaceutical Policy and Services, PPGASFAR, Universidade Federal de Santa Catarina – Campus Trindade, Florianópolis, Santa Catarina CEP 88.040-000 Brazil

**Keywords:** Rare diseases, Access to medicines, Health policy, Health technologies, Brazil

## Abstract

**Background:**

Rare diseases affect a small number of people compared to prevalent diseases. The vast majority of these diseases are of genetic origin, have no cure, are chronic and can lead to death. Although the right to access medicines is included in the constitutionally guaranteed right to health in Brazil, problems in the supply of medicines for rare diseases are reported in the country. This study aimed to describe and analyse the initiatives to promote access to medicines for treating rare diseases in the Unified Health System, Brazil, after the publication of the Brazilian Policy on the Comprehensive Care of People with Rare Diseases. Based on the model published by the WHO Regional Office for Europe, which described access to medicines in prelaunch, perilaunch and postlaunch policies, the initiatives referring to each category were summarized based on documentary research searched in online databases from January 2014 to December 2020.

**Results:**

Different actions and policy interventions were identified, which went through the expansion of resources for research and development, health regulations, incorporation of new drugs, review and publication of clinical guidelines, and expansion of the network of care facilities by the Ministry of Health. On the other hand, aspects related to care policies, pricing methods, technological development, and development of pharmaceutical service processes were not implemented.

**Conclusions:**

Although it is impossible to determine the explicit motivation of such actions concerning the Policy, its publication certainly was a landmark in Brazilian society, allowing greater recognition of the needs of rare disease patients and the specificities of treatment’. However, this study suggests that the steps that make up the life cycle of medicines are not linked, lacking articulation and integration of the care network, and consequently, there is no evidence that rare disease policy publication has generated a broad impact on the promotion of access to medicines to treat rare diseases in Brazil.

**Supplementary Information:**

The online version contains supplementary material available at 10.1186/s13023-023-02881-5.

## Background

Rare diseases affect a small number of people compared to prevalent diseases. However, there is no global consensus regarding their definition, and the prevalence threshold is the most commonly used descriptor [1].

Individually, these diseases have a low prevalence rate; on the other hand, collectively, they affect a substantial proportion of the population. For example, a recent study based on the European definition of rare diseases estimated between 6,000 and 8,000 types of rare diseases that affect at least 3.5–5.9% of the world population; 71.9% of those diseases are genetic and inherited, and 69.9% begin to manifest during childhood [2]. The referenced study excluded rare cancers, infectious diseases, and poisoning disorders.

In 2014, Brazil published a rare disease policy stating that rare diseases affect up to 65 people per 100,000 inhabitants [3].

The Brazilian Policy on the Comprehensive Care of People with Rare Diseases (BPCCPRD) is organized in structuring axes according to the common characteristics of the diseases: Axis I includes rare genetic diseases, and Axis II includes rare nongenetic diseases. Among the principles of the BPCCPRD are the incorporation and use of technologies aimed at promotion, prevention, and comprehensive care in the Health Care Network (HCN). Such care includes medicinal treatment and nutritional formulas when recommended within the scope of the Brazilian Unified Health System (Portuguese: Sistema Único de Saúde, SUS), whose incorporations should result from the recommendations made by the National Committee for Health Technology Incorporation (CONITEC) and the subsequent drafting of clinical guidelines [3, 4]. In the SUS, medicines for the treatment of rare diseases are mostly made available through the Specialized Component of Pharmaceutical Services (CEAF) strategy, free of charge for patients. The funding and procurement of these medicines are agreed upon among the union, states, and municipalities, organized by a commission called the Tripartite Interagency Commission (CIT). Group “1” CEAF medicines are procured and financed by the Ministry of Health. Group “2” CEAF medicines are the responsibility of the Ministry of Health and state governments. Medicines in Group 3 are the responsibility of municipalities [5].

Furthermore, the BPCCPRD proposed two complementary structural components to the HCN: the Specialized Healthcare Center (Portuguese: Serviço de Atenção Especializada) and the Rare Diseases Reference Center (Portuguese: Serviço de Referência em Doenças Raras). It also created financial incentives for accredited centres and incorporated genetic and biochemical tests [3].

In the SUS, pharmaceutical services pose a considerable challenge for managers and professionals, mainly due to the financial resources involved and the need for technical and organizational improvement. On the one hand, advances in structuring public health policies have allowed greater access to medicines and SUS services; on the other hand, there is still a need for continuous improvements, mainly regarding the population with specific needs. For example, many sufferers from rare diseases face barriers in accessing care, and a decade ago, less than 10% received specific treatment for the diseases, according to estimates [6].

In low- or middle-income countries, such as Brazil, the availability in the market of medicines for treating rare diseases does not guarantee that patients will have access to them, given the gaps in the country’s integrated solutions to ensure universal health coverage [7]. Some of these gaps pointed out by authors are limited resources and accessibility [8, 9], technological dependence [7], the inadequacy of metrics in Health Technology Assessments (HTAs) [10], the non-incorporation of technology into the public health system [11], and excessive market prices [12, 13].

According to the Brazilian Constitution, access to medicines can be understood as part of the right to health. However, problems in medicine provision are broadly reported [8, 14], and access to treatment is only possible via legal proceedings [8,14.

In this sense, this study aimed to describe and analyse the initiatives to promote access to medicine for treating rare diseases in the SUS after the publication and implementation of the BPCCPRD.

## Methods

Brazilian government initiatives were summarized considering the WHO model for access to new medicines [15]. The proposed model subdivides medicine access policies into prelaunch, prelaunch, and postlaunch activities. The governmental reports selected are categorized in Table 1.

**Table 1:** Categories of analyses based on WHO guidelines

Category	Definition	Interventions analysed
Prelaunch	Includes activities to anticipate and prioritize therapeutic innovation with potential impacts on the health system and clinical outcomes	research and development, clinical trials, and horizon scanning
Prelaunch	Includes policies to manage the entry of new medicines into the health system	pricing methods, regulatory mechanisms for marketing authorization, managed entry agreements, incorporation of medicines into the health system
Postlaunch	Includes policy requirements to ensure access to and the use of medicines in the health system	procurement, distribution, prescribing, clinical guidelines, dispensing, and pharmacovigilance activities

The analysed data are in the public domain and were researched through a documentary assessment of online databases previously defined by the researchers. The sources and searches performed are described in Additional files 1 and 2. The results from this current evidence were summarized using a narrative synthesis and tables.

### Prelaunch

#### Scientific research and clinical trials

Clinical trials and scientific research related to medicines or nutritional supplementation to treat rare diseases conducted between 2014 and 2020 were searched. To this end, all clinical trial registries were searched in the Brazilian Registry of Clinical Trials (ReBEC). After individual surveys, all studies involving new medicine research or improvement/assessment of existing treatments were selected.

On Plataforma Brasil, a search for research involving developing medicines and/or nutritional formulas for rare diseases was performed using the keyword “rare diseases” (in Portuguese and English, singular and plural). On ClinicalTrials.gov, a search was performed among all studies registered in Brazil with the keyword “rare diseases”.

#### Horizon scanning

A systematic search of the CONITEC website (Additional file 1) was conducted to identify a publication of information related to new and emerging technologies. On the database, horizon scanning was described in two categories: alerts, which signal a particular new and emerging technology, and reports, which track new and emerging technologies in a specific area.

### Perilaunch

#### Rare disease medicines authorized in the country

A descriptive and exploratory study was conducted of the National Health Surveillance Agency (ANVISA) website.

In the database, a search was conducted for medicines registered in the following regulatory categories: biological (medicines that contain a molecule with known biological activity) [16]; new (medicines with a new active pharmaceutical ingredient in the country) [17]; advanced therapy products (advanced cellular therapy products, tissue engineering products, gene therapy products) [18]; and specific (technically obtained or prepared pharmaceutical products for prophylactic, curative or palliative purposes, regardless of nature or origin and not susceptible to bioequivalence testing against a comparator product) [19].

#### Rare disease medicines in the SUS

A descriptive and exploratory study of the CONITEC recommendations for medicines and clinical guidelines to treat rare diseases was conducted between January 2014 and December 2020. The rare diseases considered for this data collection were those listed by Cunico and Leite [20].

When the technology was indicated more than once for different therapeutic indications or when a single submission presented two different technologies, they were quantified individually. The recommendations were evaluated according to the active ingredient, clinical indication, request agreement (incorporating or not incorporating), and applicant type (internal: bodies or institutions linked to the SUS; external: bodies or institutions not linked to the SUS, or individual).

The time elapsed to make the medication available at the SUS was evaluated. First, the time elapsed between proposal submission and recommendation decision publication was evaluated. Then, the time elapsed between the recommendation decision for incorporation and the publication of the clinical guideline was evaluated.

The technologies were categorized according to the recommendation decision publication year, with the axes defined by the BPCCPRD.

#### Responsibility for financing medicines for rare diseases in the SUS

The SUS management sphere responsible for financing each medicine on the list of incorporated medicines for rare diseases between January 2014 and December 2020 was identified. Searches of the agendas and summaries of the CIT meetings and the Brazilian National List of Medicines were conducted [21].

Additionally, we assessed the time elapsed between CONITEC publication of the incorporation decision and agreement on financing responsibility at the CIT.

### Postlaunch

#### Partnerships for productive development

The listings of strategic products to the SUS eligible for submitting proposals for Partnership for Productive Development (PDP) projects in 2014–2020 were searched. In addition, the listing of products with partnerships in force in December 2020 was searched and is described in this study.

#### National Reference Services for Rare Diseases

Based on the code system provided by the legislation, the accredited services to people with rare diseases within the National Register of Health Facilities were searched. Data concerning the geographic location and types of rare diseases treated for each accredited service were described.

## Results

Different initiatives were identified in the prelaunch, perilaunch, and postlaunch categories from 2014 to 2020.

### Prelaunch

The establishment of the National Policy for Technological Innovation in Health, regulatory procedures related to research involving human beings, and clinical trials with medicines were identified. Furthermore, the methods by which the industry could offer experimental medicines to patients suffering from diseases hitherto untreated in the country were defined, and the right to poststudy access to clinical research protocols was granted to patients with ultrarare diseases.

The search on research registration platforms identified some studies involving medicines. In ReBEC, most of the trials registered referred to therapeutic alternatives for medicines already in use in the country, with the involvement of higher education institutions and publicly traded corporations/private legal entities. On the other hand, research with new medicines had the pharmaceutical industry as the entity responsible for the study and involvement of private equity (Additional file 3).

Moreover, in 2019, the National Council for Scientific and Technological Development (CNPq), in partnership with the Ministry of Health of Brazil, issued a call for research to obtain epidemiological information on rare diseases in Brazil [22].

The promotion and incentives for research on rare diseases are goals of the federal public administration and the work of the Interministerial Committee on Rare Diseases. In addition, 30% of the resources destined for the Health Research Promotion Program for the technological development of medicines, immunobiological products, health products, and other therapeutic modalities are now allocated to treat rare or neglected diseases (Additional file 2).

Horizon scanning is being improved in the country resulting from a partnership between the Ministry of Health of Brazil and the Biomedical Engineering Program of the Alberto Luiz Coimbra Institute for Graduate Studies and Research in Engineering (COPPE) of the Federal University of Rio de Janeiro and the Support Program for the Institutional Development of the Brazilian Public Health System (PROADI-SUS) [23].

Furthermore, a systematic search on the CONITEC website showed that four alerts (2 in 2016, 1 in 2017, and 1 in 2018), three reports (2017, 2019, 2020), and 19 sections in recommendation reports of studied medicines (9 in 2018, 6 in 2019, and 4 in 2020) were published while this study was being conducted.

### Perilaunch

In Brazil, the sanitary registration of medicines for rare diseases was standardized in 2017 with the publication of the Collegiate Board Resolution (RDC) 205 [24] and its following updates. The swiftness in regulatory processes for rare disease medicines and the regulatory bases for registering new cellular therapy products and human gene therapy products were also regulated (Additional file 2).

Thirty-seven rare disease medicines were identified in the following regulatory categories: biological (n = 21); new (n = 13); advanced therapy products (n = 2); and specific (n = 1) (Table 2).

**Table 2:** Medicines for rare diseases registered after RDC^a^ 205/2017, ANVISA^b^, Brazil, 2017–2020

Medicines	Dosage	Clinical indication	Registration date	Registration validity	Regulatory category	Demanded to SUS^c^/incorporated into SUS
Ocrelizumab	30 mg/mL	Multiple sclerosis	02/26/2018	Feb/28	Biological	Yes/No
Nonacog Gamma	250 UI; 500 UI; 1000 UI; 2000 UI; 3000 UI	Hemophilia B	02/26/2018	Aug/29	Biological	No/No
Agalsidase Beta	35 mg/20 mL	Fabry disease	04/30/2018	Apr/20	Biological	Yes/No
Cerliponase Alfa	30 mg/mL	Neuronal ceroid lipofuscinosis type 2	07/16/2018	Jul/28	Biological	No/No
Emicizumab	30 mg/mL; 60 mg/0.4 mL; 105 mg/0.7 mL; 150 mg/mL	Hemophilia A	07/16/2018	Jul /28	Biological	Yes/Yes
Ivacaftor + Lumacaftor	125 mg + 100 mg; 125 mg + 200 mg	Cystic fibrosis	07/23/2018	Jul /23	New	Yes/No
Ivacaftor	150 mg	Cystic fibrosis	09/03/2018	Sep/23	New	Yes/Yes
Albutrepenonacog Alfa	250 UI; 500 UI; 1000 UI; 2000 UI;	Hemophilia B	10/08/2018	Oct/28	Biological	No/No
Vestronidase Alfa	10 mg/5 mL	Mucopolysaccharidosis VII	10/15/2018	Oct/28	Biological	Yes/Yes
Defibrotide	80 mg/mL	Liver veno-occlusive disease	03/11/2019	Mar/29	Biological	No/No
Burosumab	20 mg/mL	X-linked hypophosphatemia in adults and children (age > 1 year old)	03/25/2019	Mar/29	Biological	Yes/No
Ataluren	125 mg; 250 mg; 1000 mg	Duchenne muscular dystrophy	04/29/2019	Apr/29	New	No/No
Selexipag	0.2 mg; 0.4 mg; 0.6 mg; 0.8 mg; 1 mg; 1.2 mg; 1.6 mg	Pulmonary arterial hypertension	04/29/2019	Jan/28	New	Yes/Under review
Carglumic acid	200 mg	Hyperammonemia	06/10/2019	Jun/29	New	No/No
Hemin	350 mg	Acute intermittent porphyria	08/19/2019	Aug/29	Biological	No/No
Ravulizumab	10 mg/mL	Paroxysmal nocturnal hemoglobinuria	09/02/2019	Sep/29	Biological	No/No
Betaine	1 g/g	Homocystinuria	09/09/2019	Sep/24	Specific	No/No
Nitisinone	2 mg; 5 mg; 10 mg; 20 mg	Hereditary type 1 tyrosinemia	10/07/2019	Oct/24	New	No/No
Inotersen	200 mg/mL	Polyneuropathy caused by hereditary transthyretin-mediated amyloidosis	10/29/2019	Oct/24	New	No/No
Migalastat	123 mg	Fabry disease	12/02/2019	Dec/24	New	Yes/under review
Lonoctocog Alfa	250 UI; 500 UI; 1000 UI; 2000 UI; 3000 UI	Hemophilia A	12/02/2019	Dec/29	Biological	No/No
Octocogue Beta	250 UI; 500 UI; 1000 UI; 2000 UI; 3000 UI	Hemophilia A	01/06/2020	Jan/30	Biological	No/No
(Tezacaftor/Ivacaftor) + Ivacaftor	(100 mg/150 mg) + 150 mg	Cystic fibrosis	01/27/2020	Jan/23	New	No/No
Damoctocog Alfa Pegol	500 UI; 1000 UI; 2000 UI; 3000 UI	Hemophilia A	02/17/2020	Feb/30	Biological	Yes/under review
Patisiran	2 mg/mL	Polyneuropathy in hereditary transthyretin-mediated amyloidosis (hATTR amyloidosis)	02/26/2020	Feb/23	New	No/No
Crizanlizumab	10 mg/mL	Vaso-occlusive crisis in sickle cell anemia	03/02/2020	Mar/23	Biological	No/No
Velmanase Alfa	10 mg	Alpha-mannosidosis	04/06/2020	Apr/23	Biological	No/No
Givosiran	189 mg/mL	Acute hepatic porphyria in adults	07/20/2020	Jul/23	New	No/No
Voretigene Neparvovec	5.0 × 10^12^ gv/mL^d^	Inherited retinal dystrophies	08/06/2020	Aug/25	Advanced Therapy Products	No/No
Onasemnogene Abeparvovec-xioi	2.0 × 10^13^ gv/mL	Spinal muscular atrophy (SMA)	08/17/2020	Aug/25	Advanced Therapy Products	No/No
Rurioctocog Alfa Pegol	250 UI; 500 UI; 750 UI; 1000 UI; 1500 UI; 2000 UI; 3000 UI	Hemophilia A	09/28/2020	Jan/30	Biological	Yes/under review
Teduglutide	5 mg	Short bowel syndrome	09/28/2020	Nov/28	Biological	No/No
Agalsidase Alfa	1 mg/mL	Fabry disease	09/28/2020	Jul/29	Biological	Yes/No
Lanadelumab	150 mg/mL	Hereditary angioedema (HAE)	09/28/2020	Oct/29	Biological	Yes/under review
Risdiplam	0.75 mg/mL	Spinal muscular atrophy (SMA)	10/13/2020	Oct/23	New	Yes/under review
Lomitapide	5 mg; 10 mg; 20 mg	Homozygous familial hypercholesterolemia	12/07/2020	Dec/23	New	No/No
Satralizumab	120 mg/mL	Neuromyelitis optica spectrum disorder	12/21/2020	Dec/30	Biological	No/No

The search corresponds to the period 12/01/2017 to 31/2020

^a^RDC = Collegiate Board Resolution

^b^ANVISA = National Health Surveillance Agency

^c^SUS = Brazilian Health System

^d^gv/mL = viral genome/mL

*Source*: Prepared by the authors based on data from the digital repositories of the National Health Surveillance Agency (ANVISA) and National Committee for Health Technology Incorporation (CONITEC)

Regarding HTAs, no specified or validated criteria for rare diseases was identified. The Brazilian Network for Health Technology Assessment (REBRATS) held several events: a discussion on multicriteria decision analysis (MCDA) in HTAs; a workshop in partnership with the National Institute for Health and Care Excellence (NICE) to address methodological aspects of HTAs and strategies for the involvement of patients, industry sectors, and other stakeholders; and a congress on patient involvement in the HTA process.

In October 2020, CONITEC launched on its website an area designed for public and patient involvement called Patient’s Perspective (Portuguese: Perspectiva do Paciente), where patients or caregivers can write about their experiences concerning the disease they are facing. Additionally, rare diseases were the subject of two public calls for medicine analysis between October and December 2020.

This study identified 64 incorporation requests of medicines for rare diseases. External applicants submitted most proposals (n = 41, 64.1%), and the Ministry of Health submitted 23 requests (35.9%). There was agreement in the recommendations in 32 decisions (56.2% from external applicants and 43.8% from internal applicants). The incorporation or non-incorporation recommendations are described in Table 3.

**Table 3:** Recommendations obtained from CONITEC according to the year of publication, Brazil, 2014–2020

Year	Clinical indication	Medicines	Recommendation	Financing group**
Rare genetic diseases (Axis I)*				
2014	Gaucher disease	Alfataliglicerace	Incorporation	1A
2015	Hereditary angioedema	Icatibant	Non-incorporation	
	Sickle cell disease	Erythropoietin	Non-incorporation	
	Congenital adrenal hyperplasia	Hydrocortisone 10 mg	Incorporation	2
	Congenital adrenal hyperplasia	Hydrocortisone 20 mg	Incorporation	2
2016	Cystic fibrosis	Tobramycin	Incorporation	1A
2017	Hemophilia B (age < 19 years old)	Nonacog Alfa	Non-incorporation	
	Mucopolysaccharidosis I	Laronidase	Incorporation	1A
	Mucopolysaccharidosis II	Idursulfase	Incorporation	1A
2018	Fabry disease	Agalsidase Alfa	Non-incorporation	
	Fabry disease	Agalsidase Beta	Non-incorporation	
	Phenylketonuria	Sapropterin	Incorporation	1B
	Paroxistic Nocturnal Hemoglobinuria	Eculizumab	Incorporation	1A
	Homozygous Familial Hypercholesterolemia	Evolucumab	Non-incorporation	
	Mucopolysaccharidosis IVa	Elosulfase Alfa	Incorporation	1A
	Mucopolysaccharidosis VI	Galsulfase	Incorporation	1A
	Familial Amyloid Polyneuropathy	Tafamidis	Incorporation	1A
2019	5q spinal muscular atrophy—type 1*	Nusinersen	Incorporation	1A
	Pompe disease	Alglucosidase Alfa	Incorporation	1A
	Niemann–Pick type C disease	Miglustat	Non-incorporation	
	Paroxysmal nocturnal hemoglobinuria	Meningococcal Conjugate Vaccines ACWY	Incorporation	***
	Paroxysmal nocturnal hemoglobinuria	Meningococcal B Vacines (Recombinant)	Non-incorporation	
	Hemophilia A with factor VIII inhibitor	Emicizumab	Incorporation	***
	Hemophilia A	Eftrenonacog Alfa	Non-incorporation	
	Hemophilia B	Eftrenonacog Alfa	Non-incorporation	
	Classical homocystinuria	Methionine-free metabolic formula	Incorporation	****
	Atypical hemolytic uremic syndrome	Eculizumab	Non-incorporation	
2020	Cystic fibrosis	Ivacaftor	Incorporation	****
	Congenital hypothyroidism	Levothyroxine	Incorporation	3
	Mucopolysaccharidosis VII	Vestronidase Alfa	Incorporation	1A
Rare nongenetic diseases (Axis II)*				
2014	Multiple sclerosis	Fingolimod	Incorporation	1A
2015	Acromegaly	Pegvisomant	Non-incorporation	
2016	Ankylosing spondylitis	Golimumab	Incorporation	1A
	Relapsing–remitting multiple sclerosis	Dimethyl Fumarate	Non-incorporation	
2017	Crohn’s disease	Certulizumab	Incorporation	1A
	Relapsing–remitting multiple sclerosis	Fingolimod	Incorporation	1A
	Relapsing–remitting multiple sclerosis	Dimethyl Fumarate	Incorporation	****
	Relapsing–remitting multiple sclerosis	Teriflunomide	Incorporation	1A
	Relapsing–remitting multiple sclerosis	Aletumzumab	Non-incorporation	
2018	Primary biliary cholangitis	Ursodeoxycholic Acid	Incorporation	1B
	Paget’s disease	Zoledronic Acid	Incorporation	****
	Relapsing–remitting multiple sclerosis	Glatiramer 40 mg	Incorporation	1A
	Ankylosing spondylitis	Secukinumab	Incorporation	1A
	Idiopathic thrombocytopenic purpura	Eltrombopag Olamine	Incorporation	1B
	Non-infectious intermediate uveitis, posterior uveitis and active panuveitis	Adalimumab	Incorporation	1A
	Relapsing–remitting multiple sclerosis	Alentuzumab	Non-incorporation	
	Idiopathic pulmonary fibrosis	Nintedanib	Non-incorporation	
	Idiopathic pulmonary fibrosis	Pirfenidone	Non-incorporation	
	Inoperable chronic thromboembolic pulmonary hypertension	Riociguat	Non-incorporation	
	Systemic lupus erythematosus	Belimumab	Non-incorporation	
	Idiopathic thrombocytopenic purpura	Romiplostim	Non-incorporation	
	Non-infectious intermediate uveitis, posterior uveitis and inactive panuveitis	Adalimumab	Non-incorporation	
2019	Relapsing–remitting multiple sclerosis	Dimethyl Fumarate	Incorporation	****
	Juvenile idiopathic arthritis	Canakinumab	Non-incorporation	
	Crohn’s disease	Vendolizumab	Non-incorporation	
	Relapsing–remitting multiple sclerosis	Ocrelizumab	Non-incorporation	
	Progressive multiple sclerosis	Ocrelizumab	Non-incorporation	
	Inoperable chronic thromboembolic pulmonary hypertension or recurrent after surgery	Riociguat	Non-incorporation	
	Ankylosing spondylitis	Secukinumab	Non-incorporation	

*Axis according to BPCCPRD, Ordinance nº 199, 2014 [3]

**Group 1A: medicines are procured and financed by the Ministry of Health. Group 1B: medicines are the responsibility of the Ministry of Health and state governments. Group 2: medicines are the responsibility of state governments. Group 3: medicines are the responsibility of municipalities

***In Brazil, vaccines and blood components are considered strategic inputs for the SUS care, and belong to the Strategic Component of Pharmaceutical Assistance (CESAF) strategy: they are financed, purchased, and distributed centrally by the Ministry of Health

****Data were not available at the time of this study

*Source*: Prepared by the authors based on data from the digital repositories of the National Committee for Health Technology Incorporation (CONITEC)

To obtain the 32 incorporation recommendations, the time elapsed to achieve a result was 233 days (median; n = 32; minimum time = 49 days; maximum time = 607 days). In Brazil, legislation [25] establishes a deadline of 180 days (which can be extended by 90 days) for completion of the health technology assessment process in the SUS.

In Brazil, in 2018, the recommendations to incorporate some medicines for rare diseases (nusinersen, eculizumab, galsulfase, elosulfase alfa) started to require patient follow-up in reference centres to collect evidence of clinical effectiveness in real-world situations. Then, within three years, a subsequent reassessment by CONITEC must be conducted, which in the reports is called *ad experimentum* use reports.

An innovative approach to financing the purchase occurred in April 2019, when the Ministry of Health decided to incorporate nusinersen for spinal muscular atrophy types 2 and 3 through a risk-sharing agreement. This project was an alternative measure to produce additional evidence about the therapeutic value of these medicines and observe the real impacts on patients’ health and quality of life. However, this agreement failed due to the lack of detailed regulations and legal standards for implementation within the SUS scope [26].

A bill related to the cost and supply of medicines and therapies for treating rare or neglected diseases is in progress in the Chamber of Deputies and awaits conclusive consideration (Additional file 1).

### Postlaunch

CONITEC made public and patient involvement possible through a poll (Portuguese: Enquete) to discuss aspects in the initial phase of clinical guideline (Portuguese: Protocolos Clínicos e Diretrizes Terapêuticas (PCDT)) drafting. During this study, four polls addressed rare diseases.

In the study period, 31 PCDTs for rare diseases were updated, and 16 new PCDTs were implemented, for a total of 51 PCDTs.

After the recommendation for incorporating the medicines into the SUS, the technical areas have 180 days to provide the population with the medicines. Establishing clinical protocols is one of the requirements for access [25].

In investigating the time elapsed between the decision to recommend incorporation and the publication of the clinical guideline for the 11 rare diseases for which there was no prior treatment available in the SUS and whose medicines were recommended during the study period, eight protocols did not comply with the period established by law. Thus, the data obtained in this study were for 292 days (median; n = 11; minimum time = 156 days; maximum time = 351 days).

Regarding the responsibility for financing, most of the medicines incorporated for rare diseases are procured and financed by the Ministry of Health (Group 1A CEAF). Table 3 describes the funding groups for the medicines recommended for incorporation.

When assessing the period required for the financial agreement, in 15 situations, the timeframe did not comply with the law [25]. The data obtained in this study were for 193 days (median; n = 24; minimum time = 10 days; maximum time = 778 days).

For national public medicine manufacturing, as of 2015, some medicines for treating rare diseases were on the SUS list of strategic products and eligible for Partnership for Productive Development (PDP) project proposal submissions. The partnerships in force in 2020 [27] included treatments for multiple sclerosis (interferon beta-1a, fingolimod 0.5 mg, teriflunomide 14 mg); juvenile idiopathic arthritis (leflunomide 20 mg); juvenile idiopathic arthritis and ankylosing spondylitis (adalimumab 40 mg/0.8 mL, certolizumab pegol 200 mg/mL, etanercept 25 and 50 mg/mL, golimumab 50 mg, infliximab 10 mg/mL, tocilizumab 20 mg/mL); sickle cell anaemia (hydroxyurea 500 mg); Crohn’s disease (infliximab 10 mg/mL); pulmonary arterial hypertension (sildenafil 20 mg, 25 mg, 50 mg); haemophilia (recombinant factor VIII); hypopituitarism and Turner syndrome (somatropin 4 IU and 12 IU); acromegaly (cabergoline 0.5 mg); Gaucher disease (taliglucerase alfa 200 UI); and amyotrophic lateral sclerosis (riluzole 50 mg).

The service offer was evaluated by identifying qualified reference centres after the BPCCPRD. By 2020, 19 reference services for rare diseases had been enabled in the country, according to the data described in Table 4. The data show a disparity in the geographical distribution of the supply of reference services among the federation states and between the capital and interior in the same state.

**Table 4:** Reference services for rare diseases, Brazil, 2014–2020

Geographic location	State	City	Initial jurisdiction	Axis I^a^	Axis II^b^
Center-West	Distrito Federal	Distrito Federal	12/2016	1, 2, 3	1,3
Center-West	Distrito Federal	Distrito Federal	12/2019	1, 2, 3	1,2,3
Center-West	Goiás	Anápolis	10/2016	1, 2, 3	1
Northeast	Pernambuco	Recife	10/2016	1, 3	1,2
Northeast	Bahia	Salvador	07/2018	1, 2, 3	
Northeast	Bahia	Salvador	06/2019	1, 2, 3	1,2,3
Northeast	Ceará	Fortaleza	12/2019	1, 2, 3	1,2,3,4
Northeast	Ceará	Fortaleza	12/2019	1, 2, 3	1,2,3,4
Southeast	Minas Gerais	Belo Horizonte	12/2019	1, 2, 3	
Southeast	Espírito Santo	Vitória	12/2019	1	
Southeast	Rio de Janeiro	Rio de Janeiro	12/2016	1, 2, 3	
Southeast	São Paulo	Campinas	12/2019	1, 2, 3	
Southeast	São Paulo	Santo André	12/2016	2, 3	2,3
Southeast	São Paulo	Ribeirão Preto	12/2019	1, 2, 3	
Southeast	São Paulo	Ribeirão Preto	12/2020	1,2,3	1,2,3
South	Paraná	Curitiba	10/2016	1, 2, 3	1,2,3
South	Paraná	Curitiba	12/2020	1	
South	Rio Grande do Sul	Porto Alegre	12/2016	1, 2, 3	
South	Santa Catarina	Florianópolis	12/2019	1, 2, 3	1,2,3

^a^Axis I = Rare genetic diseases, with three groups: 1—Congenital or late-onset anomalies, 2—Intellectual disability, 3—Inborn errors of metabolism

^b^Axis II = Rare nongenetic diseases, with the following groups of causes: 1—Infectious, 2—Inflammatory, 3—Autoimmune, 4—Other rare nongenetic diseases

*Source*: Prepared by the authors based on National Register of Health Facilities [28] Axes according to BPCCPRD

Many medicines for treating rare diseases are listed in the CEAF strategy. In 2020, a change was identified in its execution model, increasing from three to six months the expiry period of the Report for the Request, Evaluation, and Authorization of Medicines from the Specialized Component of Pharmaceutical Assistance (Portuguese: LME).

Due to the SARS-CoV-2 (severe acute respiratory syndrome coronavirus 2) emerged, special criteria for implementing the CEAF strategy related to documentation renewal and dispensing anticipation were advocated. Additionally, patients with Gaucher disease, assisted by the Ministry of Health through the provision of enzymatic therapy by Bio-Manguinhos/Fiocruz, started to be given medicine infusions via home care service [29].

Searches on the Legislative Power websites identified social demands in the scope of rare diseases, which were discussed in the Special Subcommittee on Rare Diseases of the Chamber of Deputies and in the Temporary Subcommittee on Rare Diseases of the Senate. Topics included scientific research conducted in Brazil, incorporating technologies by the SUS considering specificities, expanding diagnostic methods, defending rights and social inclusion, and improving specific legislation.

## Discussion

The analysis model chosen in this study clearly showed that a set of interconnecting policies determines access to medicines for treating rare diseases. Furthermore, this study showed that since the publication of the BPCCPRD, actions to promote access to such medicines have been developed in Brazil, including all phases of development, dispensation, and use of medicines. Although it is impossible to determine the explicit motivation of such actions concerning the BPCCPRD, its publication certainly is a landmark in Brazilian society, allowing greater recognition of the needs of rare disease patients and the specificities of treatment. Therefore, expanding and guaranteeing access to pharmacological treatment is fundamental to provide a better quality of life for people affected by a rare disease.

Among prelaunch activities, the development of research for new medicines, the expansion of financial resources forecast by the federal government, and the sanitary regulation of access to innovative treatments indicate that there are concrete initiatives to expand access. According to Gomes et al. [30], aspects related to horizon scanning have positive repercussions as an informative tool for managers to reduce the risk to the health system. Furthermore, the same authors [30] mentioned that since the beginning of horizon scanning in Brazil, the most frequent themes of internal reports, alerts, summaries, and sections in the CONITEC recommendation reports were related to rare diseases. Another favourable factor is the ongoing epidemiological inquiry because the data may help rationalize medicines based on a reliable national epidemiological forecast [31]. However, despite the implementations mentioned above, it was not possible to identify a development line with long-term research, development, or innovation planning. From the data described in Additional file 3, it was not possible to identify a clear trend of increase in the number of studies for rare disease medicines after the publication of the BPCCPRD. A similar finding was also previously described by Gadelha et al. [32] in a study on Brazil’s health economic-industrial complex. Furthermore, most of the studies identified were related to the extension of use of medicines already available in the country, which expands the therapeutic possibilities in less time for patients but which, on the other hand, allows the extension of patent terms, preventing the production of generic, similar or biosimilar medicines by public laboratories, increasing costs for the SUS [33].

Among prelaunch actions, different aspects can be considered drivers of access, such as regulating the sanitary registration of rare disease medicines, which can expand the supply of new medicines in the SUS (Table 2). In this respect, ANVISA’s RDCs 204 and 205 may shorten the market authorization time, as rare disease medicines tend to meet the criteria for accelerated procedures based on an unmet need or disease severity. According to Caetano and collaborators [34], medicine manufacturers did not request the registration of medicines for rare diseases to obtain greater gain from selling their medicines via judicialization since the absence of sanitary registration made it impossible to set the price for sale to the government. In 2018, however, the judgement of Theme 106 of general repercussion in the Supreme Court of Justice (STJ) [35] started to oblige registration on ANVISA for granting medicines not incorporated into the SUS. It is worth noting that expanding the health records of medicines can expand the possibilities of prescription in the country and, when there is no inclusion in the SUS payment table, often expand accessibility through lawsuits [34].

There is a growing demand for incorporating rare disease medicines both by SUS internal and external requests, as also described by Biglia et al. [36]. Despite the increasing demand for incorporations and growth in the public and patient involvement in the HTA process, the nonadoption of methodologies that consider broader approaches or values, in addition to cost-effectiveness, can be regarded as limiting access in HTA settings for rare diseases [12, 37]. Studies describe that standardized HTA methods may have limitations when assessing these medicines, as they may not accurately measure the social value of some health technologies [10, 12]. Another limitation refers to issues related to pricing in Brazil. It is suggested that pharmaceutical industries have been applying strategies to delay the launch of these medicines in lower-income countries due to the widespread use of international pricing policy [13]. When reassessing pricing or management contracts is lacking, the growth of rare disease medicine incorporation into the health system sparks debates on the right to health [19]. Caetano et al. [38] also discussed conditional incorporation (*ad experimentum*) as a possibility for reassessing better technological alternatives based on evidence; however, the country does not have structural conditions (such as clinical data information systems and reference centres) for its successful development.

In postlaunch interventions, the reassessment of norms related to good health practices and pharmacovigilance of medicines and updates to clinical guidelines are essential actions to promote the proper and efficient use of these medicines. Furthermore, the Rare Diseases Reference Centres (Table 4), accredited by the Ministry of Health, plus the incorporated molecular biology tests, cytogenetics, immunoassays, and genetic counselling, can expand diagnostic capability and the prospect of adequate treatment to the patient at the earliest opportunity.

As already found by Mayrides et al. [39] and described in this study in Table 4, most reference centres in Brazil focus on congenital disabilities, congenital errors of metabolism, and/or intellectual disabilities. Moreover, despite the increase in the number of these services in the national territory, there are still regions lacking specialized services [39].

There was also no update in the financial resources for funding passed on to Rare Diseases Reference Centres. In addition, Brazil’s data systematization should be discussed in managing rare diseases. In Brazil, there was no development of a system to register clinical data, which hindered the integration between the different components of the HCN. According to Félix et al. [31], the Rare Diseases Reference Centres are under development. It is unknown whether the human and technological resources of these services are adequate and sufficient to achieve the care goals established by the BPCCPRD [31].

Within the scope of national manufacturing, PDPs reduce the acquisition cost of medicines that are currently imported. They also boost local manufacturing involving technology transfer to the Brazilian market [27]. Nevertheless, according to D’Ippolito and Gadelha [7], the national productive base has weaknesses that might contribute to a poorly inclusive development model [7].

Logistical issues remained unchanged. The processes of planning, acquisition, distribution network improvement and expansion of the Secretary of State for Health’s role in promoting the rational use of medicines need broader discussions to avoid gaps in patient treatment [40]. Noncompliance with the deadlines regulated by current legislation [25] for the effective supply of medicines in the SUS demonstrates that nonintegrated processes, procedures that are not well established, and insufficient human resources can hinder the operationalization and reorganization of the system [41].

The lack of alignment between the actions for access to medicines implies the occurrence of judicialization. Consequently, it burdens the system and may restrict the support of health needs while not ensuring adequacy in the administration of medicines, continuity of treatment, and follow-up by a trained health team in addition to favouring particular groups over others [42, 43].

According to Gammie et al. [44], the national guidelines for rare diseases aim to create a regulatory framework for access to services, treatment, information, encouragement of research, and patient advocacy. Nevertheless, often, they do not implement legislation for access to medicines. Legislation on orphan medicines in countries, such as the Orphan Drugs Act, passed in 1983 in the United States or Regulation (EC) No. 141/2000 in the European Union, could improve access to these medicines, according to the authors [44].

This study has severe limitations related to the perilaunch phase, since it was not possible to perform a deeper analysis of the impact of the study being financed by public, private, or mixed capital, having a national or international scope. We performed only a simple search to identify the presence or absence of clinical research initiatives in the country. Another limitation considered is that the material consulted does not present data related to the effective offer of medicines to patients, so future investigations on effective access are pertinent.

## Conclusions

The enactment of the BPCCPRD acknowledged the importance of discussing rare diseases in Brazil. The BPCCPRD also created a regulatory framework for service accessibility, increasing the demands for medicine incorporation, PCDT updates, and licensing of reference centres. However, these efforts were not aligned with care policies, pricing methods, technological development, or process management in pharmaceutical assistance, lacking articulation in the care network. Consequently, there is no evidence that its publication has generated a broad impact on promoting access to medicines to treat rare diseases in Brazil. Furthermore, it is believed that integrating the life cycle stages and usage cycle stages of medicines can improve the quality and speed of the processes, thus avoiding progressive losses in the quality of life of patients suffering from a rare disease and contributing to comprehensive care. Finally, this can also prevent increased judicialization related to medicine access.

### Supplementary Information

**Additional file 1:** Search about prelaunch, perilaunch and postlaunch activities to promote access to medicines for treating rare diseases, Brazil, 2014–2020.

**Additional file 2:** Regulations, norms or guidelines related to rare diseases after publication and implementation of the BPCCPRD, Brazil, 2014–2020.

**Additional file 3:** Research and clinical trials related to medicines for treating rare diseases conducted in Brazil, 2014–2020.

## Data Availability

Not applicable.

## Abbreviations

ANVISA: National Health Surveillance Agency
BPCCPRD: Brazilian Policy on the Comprehensive Care of People with Rare Diseases
CEAF: Specialized Component of Pharmaceutical Assistance
CIT: Tripartite Interagency Commission
CNPq: National Council for Scientific and Technological Development
CONITEC: National Committee for Health Technology Incorporation
COPPE: Biomedical Engineering Program of the Alberto Luiz Coimbra Institute for Graduate Studies and Research in Engineering
HCN: Health Care Network
HTA: Health Technology Assessment
LME: Report for the Request, Evaluation, and Authorization of Drugs from the Specialized Component of Pharmaceutical Assistance
MCDA: Multicriteria decision analysis
NICE: The National Institute for Health and Care Excellence
PCDTs: Clinical guidelines
PDP: Partnership for Productive Development
RDC: Collegiate Board Resolution
PROADI-SUS: Support Program for the Institutional Development of the Brazilian Public Health System
ReBEC: Brazilian Registry of Clinical Trials
REBRATS: Brazilian Network for Health Technology Assessment
SARS-CoV-2: Severe Acute Respiratory Syndrome Coronavirus 2
SUS: Unified Health System

## References

[1] Richter T, Nestler-Parr S, Babela R, Khan ZM, Tesoro T, Molsen E, International Society for Pharmacoeconomics and Outcomes Research Rare Disease Special Interest Group (2015). Rare disease terminology and definitions—a systematic global review: report of the ispor rare disease special interest group. Value Health..

[2] Nguengang Wakap S, Lambert DM, Olry A, Rodwell C, Gueydan C, Lanneau V (2020). Estimating cumulative point prevalence of rare diseases: analysis of the Orphanet database. Eur J Hum Genet.

[3] Brasil. Ministério da Saúde. Ordinance no. 199, 2014, January 30. Establishes the Brazilian Policy on the Comprehensive Care of People with Rare Diseases, approves the Guidelines for Comprehensive Care of People with Rare Diseases within the Unified Health System (SUS) and establishes financial incentives for funding. Diário Oficial da União, 2014. Available from: http://bvsms.saude.gov.br/bvs/saudelegis/gm/2014/prt0199_30_01_2014_rep.html. Accessed 12 Oct 2022.

[4] Brasil. Comissão Nacional de Incorporação de Tecnologias no SUS. Recommendation Report no. 142: Prioritisation of protocols and therapeutic guidelines for comprehensive care of rare disease patients. 2015. Available from: https://www.gov.br/conitec/pt-br/midias/relatorios/2015/relatrio_pcdt_doenasraras_cp_final_142_2015.pdf. Accessed 2 Mar 2020.

[5] Brasil. Portaria de Consolidação nº 2, de 28 de setembro de 2017. Consolidation of the rules on national health policies of the Unified Health System. Annex XXVIII—Title IV—Treats rules for financing and execution of the Specialized Component of Pharmaceutical Care within the scope of the SUS. Available from: http://bvsms.saude.gov.br/bvs/saudelegis/gm/2017/prc0002_03_10_2017.html. Accessed 2 Mar 2020.

[6] Melnikova I (2012). Rare diseases and orphan drugs. Nat Rev Drug Discov..

[7] D’Ippolito PIMC, Gadelha CAG (2019). The treatment of rare diseases in Brazil: the judicialization and the Health Economic-Industrial Complex. Saúde Debate.

[8] Rover MRM, Peláez CMV, Faraco EM, Farias MR, Leite NL (2017). An evaluation of governance capacity of the specialized component of pharmaceutical services in Brazil. Cien Saude Colet.

[9] Giugliani L, Vanzella C, Zambrano MB, Donis KC, Wallau TKW, Costa FMD (2019). Clinical research challenges in rare genetic diseases in Brazil. Genet Mol Biol.

[10] Araújo DV. Limitations of the methods of HTA for decision to incorporate technologies for rare diseases. 2014; Supl.1:24–9. Available from: http://www.jbes.com.br/images/edicao-especial2014/jbes-especial04.pdf. Accessed 2 Mar 2020.

[11] Lima MAFD, Gilbert ACB, Horovitz DDG (2018). Treatment networks and associations of patients with rare diseases. Cien Saude Colet.

[12] Garrison LP, Jackson T, Paul D, Kenston M (2019). Value-based pricing for emerging gene therapies: the economic case for a higher cost-effectiveness threshold. J Manag Care Spec Pharm..

[13] Vogler S, Schneider P, Zimmermann N (2019). Evolution of average european medicine price: implications for the methodology of external price referencing. Pharmaco Econ Open.

[14] Trevisan LM, Nalin T, Tonon T, Veiga LM, Vargas P, Krug BC (2015). Access to treatment for phenylketonuria by judicial means in Rio Grande do Sul. Brazil Cien Saude Colet.

[15] World Health Organization - Regional Office for Europe. Access to new medicines in Europe: technical review of policy initiatives and opportunities for collaboration and research. World Health Organization, 2015. Available from: https://www.euro.who.int/__data/assets/pdf_file/0008/306179/Access-new-medicines-TR-PIO-collaboration-research.pdf. Accessed 2 Mar 2020.

[16] Brasil. Ministério da Saúde. RDC 55, de 16 de dezembro de 2010. Technical regulation that provides for the registration of new biological products and biological products and makes other provisions. Diário Oficial da União, 2010. Available from: https://bvsms.saude.gov.br/bvs/saudelegis/anvisa/2010/res0055_16_12_2010.html. Accessed 12 Oct 2022.

[17] Brasil. Ministério da Saúde. RDC 200, de 26 de dezembro de 2017. Technical regulation that establishes the criteria for the concession and renewal of the registration of medicines with synthetic and semi-synthetic active principles, classified as new, generic and similar, and makes other provisions. Diário Oficial da União, 2018. Available from: https://www.in.gov.br/materia/-/asset_publisher/Kujrw0TZC2Mb/content/id/2198623/do1-2018-01-29-resolucao-rdc-n-200-de-26-de-dezembro-de-2017--2198619. Accessed 12 Oct 2022.

[18] Brasil. Ministério da Saúde. RDC 508, de 27 de maio de 2021. Provides for the good practices in human cells for therapeutic use and clinical research, and makes other provisions. Diário Oficial da União, 2021. Available from: https://bvsms.saude.gov.br/bvs/saudelegis/anvisa/2020/rdc0508_27_05_2021.pdf. Accessed 12 Oct 2022.

[19] Brasil. Ministério da Saúde. RDC 24, de 14 de junho de 2011. Provides for the registration of specific drugs. Available from: https://bvsms.saude.gov.br/bvs/saudelegis/anvisa/2011/rdc0024_14_06_2011.pdf. Accessed 12 Oct 2022.

[20] Cunico C, Leite SN (2022). Rare diseases: proposition of a list based on the Brazilian Health System. Expert Opin Orphan Drugs.

[21] Brasil. Ministério da Saúde. Secretaria de Ciência, Tecnologia, Inovação e Insumos Estratégicos em Saúde. Departamento de Assistência Farmacêutica e Insumos Estratégicos. Relação Nacional de Medicamentos Essenciais Rename 2022. Brasília: Ministério da Saúde, 2022. Available from: https://www.gov.br/saude/pt-br/composicao/sectics/daf/rename/20210367-rename-2022_final.pdf. Accessed 12 Oct 2022.

[22] Brasil. Ministério da Ciência, Tecnologia, Inovações e Comunicações. CNPq. Call CNPq/MS/SCTIE/DECIT Nº 25/2019 - Survey on rare disease profile in Brazil. Available from: http://memoria2.cnpq.br/web/guest/chamadas-publicas?p_p_id=resultadosportlet_WAR_resultadoscnpqportlet_INSTANCE_0ZaM&idDivulgacao=9142&filtro=encerradas&detalha=chamadaDetalhada&id=47-1412-6502. Accessed 12 Oct 2022.

[23] Brasil. Ministério da Saúde. Horizon scanning in Brazil: advances and challenges Brasília: Ministério da Saúde, 2018. Available from: https://www.gov.br/conitec/pt-br/midias/radar/2018/livromht.pdf. Accessed 12 Oct 2022.

[24] Brasil. Ministério da Saúde. RDC 205, de 28 de dezembro de 2017. Provides for special procedures for the regulation of medicines for rare diseases. Available from: https://www.in.gov.br/materia/-/asset_publisher/Kujrw0TZC2Mb/content/id/1486126/do1-2017-12-29-resolucao-rdc-n-205-de-28-de-dezembro-de-2017-1486122. Accessed 12 Oct 2022.

[25] Brasil. Decreto 7.646, de 21 de dezembro de 2011. Decree 7.646, of December 21, 2011. Provides for the National Commission for the Incorporation of Technologies into the Brazilian Health System and the administrative process for the incorporation, exclusion and alteration of health technologies by the Brazilian Health System—SUS, and makes other provisions. 2011. Available from: http://www.planalto.gov.br/CCIVIL_03/_Ato2011-2014/2011/Decreto/D7646.htm. Accessed 4 Apr 2020.

[26] Ramos TM, Thomasi TZ, DuarteJúnior DP (2020). Risk-sharing agreements for the acquisition of the Spinraza^®^ drug in Brazil: new perspectives on the legal protection of patients. Cad Ibero-Amer Dir Sanit..

[27] Brasil. Ministério da Saúde. Assuntos. Parcerias para o Desenvolvimento Produtivo. Available from: https://www.gov.br/saude/pt-br/composicao/sctie/cgcis/pdp. Accessed 10 May 2021.

[28] Brasil. DATASUS [National Register of Health Facilities]. Reports. Qualifications. Code 35. Available from: http://cnes2.datasus.gov.br/Mod_Ind_Habilitacoes.asp?VEstado=00&VTipo=H. Accessed 1 May 2021.

[29] Brasil. Ministério da Saúde. Bio-Manguinhos. Notícias e Artigos. Alfataliglicerase: patients get home care during pandemic of COVID-19. Available from: https://www.bio.fiocruz.br/index.php/br/noticias/1788-alfataliglicerase-pacientes-passam-a-ter-home-care-durante-a-pandemia-da-covid-19. Accessed 1 May 2021.

[30] Gomes PTCG, Mata VE, Borges TC, Galato D (2019). Horizon scanning in Brazil: outputs and repercussions. Rev Saude Publica..

[31] Félix TM, de Oliveira BM, Artifon M (2022). Epidemiology of rare diseases in Brazil: protocol of the Brazilian Rare Diseases Network (RARAS-BRDN). Orphanet J Rare Dis.

[32] Gadelha CAG, Costa LS, Maldonado J (2012). The Economic-Industrial Health Care Complex and the social and economic dimension of development. Rev Saúde Pública..

[33] Paranhos J, Mercadante E, Hasenclever L (2020). The cost to the Brazilian Unified National Health System of the extension of drug patent terms. Cad Saúde Pública..

[34] Caetano R, Rodrigues PHA, Correa MCV, Villardi P, Osorio-de-Castro CGS (2020). The case of eculizumab: litigation and purchases by the Brazilian Ministry of Health. Rev Saude Publica..

[35] Superior Tribunal de Justiça. Recurso Especial: REsp 1.657.156 - RJ (2017/0025629–7). Available from: https://processo.stj.jus.br/repetitivos/temas_repetitivos/pesquisa.jsp?novaConsulta=true&tipo_pesquisa=T&cod_tema_inicial=106&cod_tema_final=106. Accessed 10 Aug 2021.

[36] Biglia LV, Mendes SJ, Lima TM, Aguiar PM (2021). Incorporation of drugs for rare diseases in Brazil: Is it possible to have full access to these patients?. Cien Saude Colet..

[37] Vicente G, Cunico C, Leite SN (2021). Transforming uncertainties into legitimate regulation? NICE and CONITEC agencies’ decisions on rare diseases. Cien Saude Colet..

[38] Caetano R, Hauegen RC, Osorio-de-Castro CGS (2019). The incorporation of nusinersen by the Brazilian Unified National Health System: critical thoughts on the institutionalization of health technology assessment in Brazil. Cad Saúde Pública..

[39] Mayrides M, Ruiz de Castilla E, Szelepski S (2020). A civil society view of rare disease public policy in six Latin American countries. Orphanet J Rare Dis.

[40] Rover MRM, Faraco EB, Vargaz-Peláez CM, Colussi C, Storpirits S, Farias M, Leite SN (2021). Access to high-priced medicines: inequalities in the organization and the results among Brazilian states. Cien Saude Colet..

[41] Colpani V, Kowalski SC, Stein AT, Buehler AM, Zanetti D, Côrtes G (2020). Clinical practice guidelines in Brazil—developing a national program. Health Res Policy Syst.

[42] Lopes MT, Koch VH, Sarrubi-Junior V, Galo PR, Carneiro-Sampaio MC (2018). Difficulties in the diagnosis and treatment of rare diseases according to the perceptions of patients, relatives, and health care professionals. Clinics..

[43] Piret CNS, Medeiros CRO (2018). Doenças Raras, Medicamentos Órfãos: reflexões sobre o dark side das organizações da indústria farmacêutica. Revista Brasileira de Estudos Organizacionais..

[44] Gammie T, Lu CY, Babar ZUD (2015). Access to orphan drugs: a comprehensive review of legislations, regulations and policies in 35 countries. PLoS One.

