# Supplementary material for: Initiatives to promote access to medicines after publication of the Brazilian Policy on the Comprehensive Care of People with Rare Diseases
Source: Orphanet J Rare Dis. 2023 Aug 31;18:259. doi: 10.1186/s13023-023-02881-5 (PMC10472611; doi:10.1186/s13023-023-02881-5)
Supplement: Supplementary file 1 — Additional file 1: Search about prelaunch, perilaunch and postlaunch activities to promote access to medicines for treating rare diseases, Brazil, 2014–2020. [file 13023_2023_2881_MOESM1_ESM.docx]

Additional file 1 - Searchs about prelaunch, perilaunch and postlaunch activities to promote acess to medicines for treating rare diseases, Brazil, 2014-2020.

| **Source** | **Digital Repositories** | **Searches** |
| --- | --- | --- |
|  |  |  |
| **Prelaunch** | | |
| National Health Surveillance Agency (ANVISA) | <https://www.gov.br/anvisa/pt-br/assuntos/regulamentacao/legislacao> | Regulations, standards, procedures, related to research and development, and clinical trials for medicines intended to treat Rare Diseases. |
| National Committee for Health Technology Incorporation (CONITEC) | https://www.gov.br/conitec/pt-br/assuntos/avaliacao-de-tecnologias-em-saude/monitoramento-de-tecnologias-em-saude | Horizon scanning for medicines intended to treat Rare Diseases. |
| Legislative Power | https://legislacao.presidencia.gov.br/ | Legislation about therapeutic innovation financing or improve. |
| Ministry of Health. National Health Council | http://conselho.saude.gov.br/resolucoes-cns | Regulations related to research and development, and clinical trials. |
| Brazilian Network for Health Technology Assessment (REBRATS) | [https://rebrats.saude.gov.br](https://rebrats.saude.gov.br/) | Horizon scanning for medicines intended to treat Rare Diseases. |
| Brazilian Registry of Clinical Trials (ReBEC) | https://ensaiosclinicos.gov.br/ | Clinical trials with medicines intended to treat Rare Diseases. |
| Plataforma Brasil | <https://plataformabrasil.saude.gov.br/login.jsf> | Scientific research/clinical trials with medicines intended to treat Rare Diseases. |
| ClinicalTrials.gov | https://clinicaltrials.gov/ | Clinical trials with medicines intended to treat Rare Diseases. |
| **Perilaunch** | | |
| National Health Surveillance Agency (ANVISA) | <https://www.gov.br/anvisa/pt-br/assuntos/regulamentacao/legislacao> AND  <https://consultas.anvisa.gov.br/#/medicamentos/> | Regulations, standards, procedures, related to regulatory mechanisms for marketing authorization. Search about rare diseases medicines registered in Brazil. |
| Medicines Market Regulatory Chamber (CMED) | <https://www.gov.br/anvisa/pt-br/assuntos/medicamentos/cmed> | Regulations, standards, procedures, related to pricing methods. |
| National Committee for Health Technology Incorporation (CONITEC) | http://conitec.gov.br/ | Regulations, standards, procedures, related to Health Technologies Assessment for medicines intended to treat Rare Diseases.  Recommendations for medicines to treat Rare Diseases. |
| Ministry of Health. Pharmaceutical Care. | https://www.gov.br/saude/pt-br/composicao/sctie/daf/componentes-da-assistencia-farmaceutica-no-sus | Legislation related to the availability of new medicines on the national market.  Table of Clinical Situations from Specialized Component of Pharmaceutical Care (CEAF). |
| Legislative Power | <https://legislacao.presidencia.gov.br/> e https://www.camara.leg.br/ | Legislation related to the availability of new medicines on the national market. |
| Brazilian Network for Health Technology Assessment (REBRATS) | [https://rebrats.saude.gov.br](https://rebrats.saude.gov.br/) | Improve to teaching, research, production, dissemination and use of Health Technologies Assessment in Brazil. |
| Ministry of Health. Tripartite Interagency Commission (CIT) | <https://www.gov.br/saude/pt-br/acesso-a-informacao/gestao-do-sus/articulacao-interfederativa/cit#:~:text=A%20Comiss%C3%A3o%20Intergestores%20Tripartite%20(CIT,SUDS)%20como%20um%20colegiado%20intergovernamental> | Responsible for financing each medicines that comprises the incorporated medicines for Rare Diseases list. |
| **Postlaunch** | | |
| National Health Surveillance Agency (ANVISA) | https://www.gov.br/anvisa/pt-br/assuntos/regulamentacao/legislacao | Regulations, standards, procedures, related to the distribution, storage, transportation, pharmacosurveillance of medicines for Rare Diseases. |
| National Committee for Health Technology Incorporation (CONITEC) | http://conitec.gov.br/ | New clinical guidelines for Rare Diseases and its updates. |
| Legislative Power | https://legislacao.presidencia.gov.br/ | Regulation related to the provision of health services for Rare Diseases patients. |
| Ministry of Health. Pharmaceutical Care. | https://www.gov.br/saude/pt-br/composicao/sctie/daf/componentes-da-assistencia-farmaceutica-no-sus | Regulation related to Specialized Component of Pharmaceutical Care (CEAF) strategy. |
| Ministry of Health. National Register of Health Establishments (CNESNET) | http://cnes2.datasus.gov.br/Mod_Ind_Habilitacoes.asp?VTipo=H | Reference Services for Rare Diseases enabled in Brazil. |
| Ministry of Health. Partnership for Productive Development (PDP) | https://www.gov.br/saude/pt-br/composicao/sctie/cgcis/pdp | Regulations, standards, procedures related to national public medicines manufacturing. |

Source: Elaborated by the authors
