# Supplementary material for: Initiatives to promote access to medicines after publication of the Brazilian Policy on the Comprehensive Care of People with Rare Diseases
Source: Orphanet J Rare Dis. 2023 Aug 31;18:259. doi: 10.1186/s13023-023-02881-5 (PMC10472611; doi:10.1186/s13023-023-02881-5)
Supplement: Supplementary file 2 — Additional file 2: Regulations, norms or guidelines related to rare diseases after publication and implementation of the BPCCPRD, Brazil, 2014–2020. [file 13023_2023_2881_MOESM2_ESM.docx]

Additional file 2 – Regulations, norms or guidelines related to rare diseases after publication and implementation of the BPCCPRD, Brazil, 2014-2020.

|  | **Source** | **Regulation** | **Content** |  |
| --- | --- | --- | --- | --- |
| **Prelaunch** | | | |  |
| **Research, development and clinical trials** | | |  |  |
|  | ANVISA^a^ |  |  |  |
|  |  | RDC^b^ 9, of 20/02/2015 | Regulation for the conduct of clinical trials with medicines in Brazil. |  |
|  |  | RDC 449, of 15/12/2020 | Amends Article 38, which is related to the Medicines Clinical Development Dossier; Article 71 related to inspection, both from RDC 9, of 20/02/2015. |  |
|  |  | RDC 172, of 08/09/2017 | Procedures for the import and export of goods and products intended for scientific or technological research and research involving human beings, and makes other provisions. |  |
|  |  | RDC 260, of 21/12/2018 | Provides for the rules for conducting clinical trials with investigational advanced therapy products in Brazil, and makes other provisions. |  |
|  |  | RDC 311, of 10/10/2019 | Amends the RDC 38, of 12/08/2013, which approves the regulations for expanded access programs, compassionate use and post-study medicines supply. |  |
|  |  | IN^c^ 20, of 20/10/2017 | Good clinical practice inspection procedures for clinical medicines trials. |  |
|  |  | Guidance nº 35/2020 – version 1 | Good clinical practice inspection guide for clinical trials with medicines and biological products - Inspection of Clinical Trial Centers. |  |
|  |  | Guidance nº 36/2020 – version 1 | Good clinical practice inspection guide for clinical trials with medicines and biological products - Inspection of Sponsors and Representative Clinical Research Organizations. |  |
|  | National Health Council |  |  |  |
|  |  | Resolução 563, of 10/11/2017 | Regulates the research participant rights to post-study access in clinical research protocols for patients diagnosed with ultra-rare diseases. |  |
|  |  |  |  |  |
|  | Legislative Power | |  |  |
|  |  | Decree 9245, of 20/12/2017 | Establishes the National Policy for Technological Innovation in Health. |  |
|  |  | Decree 9283, of 07/02/2018 | Establishes incentives for innovation and scientific and technological research in the production labs, aiming at technological qualification, the achievement of technological autonomy, and the development of the national and regional production system. |  |
|  |  |  |  |  |
|  |  | Law 13930, of 10/12/2019 | Amends the Law 10332, including the forecast of application of at least 30% of the resources from the Program of Incentive to Research in Health for activities directed to the technological development of medicines, immunobiologicals, health products and other therapeutic modalities destined to the treatment of rare or neglected diseases. |  |
|  |  | Law 13971, of 27/12/2019 | It institutes the Multi-Year Plan for the period from 2020 to 2023, proposing the fostering of scientific and technological research, focused on health care, including the prevention and treatment of rare diseases. |  |
|  |  | Decree 10588, of 03/12/2020 | Creates the Interministerial Committee for Rare Diseases |  |
| **Perilaunch** | | | |  |
| **Market authorization** | |  |  |  |
|  | ANVISA |  |  |  |
|  |  | RDC 205, of 28/12/2017 | Establishes special procedures for consent of clinical trials, certification of good manufacturing practices and registration of new medicines for treatment, diagnosis or prevention of rare diseases. |  |
|  |  | RDC 293, of 17/07/ 2019 | Amends Article19 of RDC 205, which deals with the deadline for the submission of the maximum price definition dossier for the submission of registration applications of new medicines. |  |
|  |  | RDC 204, of 27/12/2017 | Provides on the classification in the priority category of registration, post-registration and prior consent petitions in clinical research of medicines. Article 4 - Frames as priority the petitions related to new therapeutic indication or extension of use intended for rare diseases. |  |
|  |  | RDC 338, of 20/02/2020 | Provides for the registration of advanced therapy products and makes other provisions. |  |
| **Managed entry** | |  |  |  |
|  | Ministry of Health |  |  |  |
|  |  | Ordinance 1297, of 11/07/2019 | Establishes a risk-sharing pilot project for the incorporation of health technologies to provide access to the medicine Nusinersena (Spinraza®) for the treatment of Spinal Muscular Atrophy types II and III within the SUS. |  |
| **Payment** | |  |  |  |
|  | Legislative Power | |  |  |
|  |  | Draf Legislation^d^ 3262/2020 | Amends the Law 13,930 of 10/12/2019 and creates the National Fund for the Supply of Medicines and Therapies intended for the Treatment of Rare or Neglected Diseases. |  |
| **Dissemination of HTA** |  |  |  |  |
|  | REBRATS^e^ |  |  |  |
|  |  | Ordinance 2575, of 30/09/2019 | Provides on the collaborative nature of REBRATS in the scope of the Secretariat of Science, Technology, Innovation and Strategic Inputs in Health of the Ministry of Health, with its management under the responsibility of the Department of Management and Incorporation of Technologies and Innovation in Health of this Secretariat. |  |
| **Postlaunch** | | | |  |
| **Distribution, storage and transportation of medicines** | | | |  |
|  | ANVISA |  |  |  |
|  |  | RDC 304, of 17/09/2019 | Provides good practice for distribution, storage and transportation of medicines. |  |
| **Financig and execution** | | |  |  |
|  | Ministry of Health |  |  |  |
|  |  | Consolidation Ordinance nº 6/GM/MS^f^, of 28/09/2017 | Repealed Ordinance GM/MS 1554/2013 that provides on the Financing of the Specialized Component of Pharmaceutical Assistance (CEAF), but there is no change in its contente. |  |
|  |  | Consolidation Ordinance nº 2/GM/MS, of 28/09/2017 | Revoked Ordinance MS/GM 1554/2013 which provides on rules for Financing and Execution of the CEAF, but there is no change in its content. |  |
|  |  | Ordinance 13, of 06/01/2020 | Changes the rules of Financing and Execution in the CEAF, regarding the 6-month validity period, validity and model of the Report for Request, Evaluation and Authorization of Medicines of the Specialized Component of Pharmaceutical Assistance (LME). |  |
|  |  | Letter nº 9, nº 11, nº 17 and 37/2020/MS | Orientations about the execution of the CEAF in the COVID-19 pandemic scenario. |  |
| **Pharmacosurveillance** | |  |  |  |
|  | ANVISA |  |  |  |
|  |  | RDC 406, of 22/07/2020 | Provides on the good practices of pharmacosurveillance for Holders of Registration of Medicines for human use, and other provisions. |  |
| **Services offer** | |  |  |  |
|  | Legislative Power | |  |  |
|  |  | Decree 10174, of 13/12/2019 | Provides for the National Council on the Rights of Persons with Disabilities, as a higher body of parity, consultative and collegiate deliberation on public policies for persons with disabilities, established under the Ministry of Women, Family and Human Rights, providing for the participation of national organizations related to rare diseases. |  |
|  |  | Law 14023, of 08/07/2020 | Amended Article 3-J of Law 13979, of 06/02/2020, to consider caregivers and attendants of disabled persons, elderly persons or persons with rare diseases as professionals considered essential to the control of diseases and the maintenance of public order, during the public health emergency resulting from the coronavirus responsible for the outbreak of 2019. |  |
|  |  | Decree 10558, of 03/12/2020 | Creates the Interministerial Committee for Rare Diseases |  |
|  | Ministry of Health |  |  |  |
|  |  | Ordinance 3166, of 03/12/2019 | Qualifies health establishments as Reference Services in Rare Diseases. |  |
|  |  | Ordinance 3709, of  22/12/2020 | Enables Reference Services for Rare Diseases in the state of São Paulo. |  |
|  |  | Ordinance 3968, of  31/12/2020 | Enables Reference Services for Rare Diseases in the state of Paraná. |  |
| **Partnerships for Productive Development** | | |  |  |
|  | Ministry of Health |  |  |  |
|  |  | Ordinance 2531, of 12/11/2014, revoked by Consolidation Ordinance nº 5/GM/MS, de 28/09/2017 | Redefines the guidelines and criteria for defining the list of strategic products for SUS and the establishment of Partnership for Productive Development. Defines the criteria from the submission of processes, to the monitoring and evaluation of technologies. |  |
|  |  | Ordinance 2888, of 30/12/2014 | Defines the list of strategic products for SUS that are eligible for submission of PPD project proposals in 2015. |  |
|  |  | Ordinance 252, of 26/01/2017 | Defines the list of strategic products for SUS that are eligible for submission of PPD project proposals for the year 2017, revoking the previous one. |  |
|  |  | Ordinance 704, of 08/03/2017 | Defines the list of strategic products for SUS that are eligible for submission of PPD project proposals for the year 2017, revoking the previous one. |  |
|  |  | Decree 10001, of 03/092019 | Provides on the Productive Development Partnerships Deliberative Committee and the Technical Committee for Evaluation of Productive Development Partnerships. |  |

Source: Prepared by the authors based on data obtained from the digital repositories described in Additional file 1.

^a^ ANVISA = National Health Surveillance Agency

^b^ RDC = Collegiate Board Resolution

^c^ IN= Normative Instruction

^d^ Draf Legislation= Bill in progress in the Chamber of Deputies. Described in the table the one that was approved and awaits conclusive consideration.

^e^ REBRATS= Brazilian Network for Health Technology Assessment

^f^ GM/MS= Minister’s Office / Ministry of Health
