# Supplementary material for: Initiatives to promote access to medicines after publication of the Brazilian Policy on the Comprehensive Care of People with Rare Diseases
Source: Orphanet J Rare Dis. 2023 Aug 31;18:259. doi: 10.1186/s13023-023-02881-5 (PMC10472611; doi:10.1186/s13023-023-02881-5)
Supplement: Supplementary file 3 — Additional file 3: Research and clinical trials related to medicines for treating rare diseases conducted in Brazil, 2014–2020. [file 13023_2023_2881_MOESM3_ESM.docx]

Additional file 3 - Research and clinical trials related to medicines for treating rare diseases conducted in Brazil, 2014-2020.

| Repositories | Year of Study Registry | Rare Diseases | Study with new medicines (n) | Extension of therapeutic use (n) | Nutritional supplementation studie  (n) | Entity responsible  for the study | Funding | Coverage | Recruiting |
| --- | --- | --- | --- | --- | --- | --- | --- | --- | --- |
|  |  |  |  |  |  |  |  |  |  |
| ReBEC |  |  |  |  |  |  |  |  |  |
|  | 2014 |  |  |  |  |  |  |  |  |
|  |  | Pulmonary Arterial Hypertension |  | 1 |  | Health Service | Public/Privado | International | 51 |
|  | 2015 |  |  |  |  |  |  |  |  |
|  |  | Juvenile Idiopathic Arthritis |  | 1 |  | Pharmaceutical Industry | Private | International | 19 |
|  |  | Growth Hormone Deficiency |  | 1 |  | Pharmaceutical Industry | Private | Brazilian | 94 |
|  |  | Fabry Disease | 1 |  |  | Higher Education Institutions | Private | International | NR* |
|  | 2016 |  |  |  |  |  |  |  |  |
|  |  | Acromegaly |  | 1 |  | Higher Education Institutions | Public | Brazilian | 30 |
|  |  | Sickle Cell Disease |  | 1 |  | Health Service | Public/Private | Brazilian | 20 |
|  |  | Cystic Fibrosis |  |  | 1 | Higher Education Institutions | Public | Brazilian | 50 |
|  |  | Pulmonary Arterial Hypertension |  | 1 |  | Pharmaceutical Industry | Private | International | 3 |
|  | 2017 |  |  |  |  |  |  |  |  |
|  |  | Sickle Cell Disease |  |  | 1 | Higher Education Institutions | Public | Brazilian | 50 |
|  | 2018 |  |  |  |  |  |  |  |  |
|  |  | Sickle Cell Disease |  | 1 |  | Health Service | Public/Private | Brazilian | 344 |
|  |  | Mucopolysaccharidosis I | 1 |  |  | Health Service | Public/Private | Brazilian | 5 |
|  |  | Hereditary Spastic Paraparesis | | 1 |  | Higher Education Institutions | Public/Private | Brazilian | 54 |
|  | 2019 |  |  |  |  |  |  |  |  |
|  |  | Systemic Sclerosis |  | 1 |  | Private Civil Scientific Association | Public/Private | Brazilian | 40 |
|  |  | Cystic Fibrosis |  |  | 1 | Higher Education Institutions | Private | Brazilian | 80 |
|  |  | Cystic Fibrosis |  | 1 |  | Higher Education Institutions | Public/Private | Brazilian | 50 |
|  |  | Pulmonary Arterial Hypertension |  | 1 |  | Higher Education Institutions | Public/Private | Brazilian | 38 |
|  |  | Classical Homocystinuria |  |  | 1 | Higher Education Institutions | Public/Private | Brazilian | 10 |
|  | 2020 |  |  |  |  |  |  |  |  |
|  |  | Acromegaly |  | 1 |  | Health Service | Public | Brazilian | 9 |
|  |  | Cystic Fibrosis |  |  | 1 | Higher Education Institutions | Public/Private | Brazilian | 36 |
|  |  | Hemophilia A |  | 1 |  | Higher Education Institutions | Public | Brazilian | 100 |
|  |  |  |  |  |  |  |  |  |  |
| Plataforma Brasil |  |  |  |  |  |  |  |  |  |
|  | 2019-20 |  |  |  |  |  |  |  |  |
|  |  | Pompe Disease | 1 | 1 |  | NR | NR | NR | NR |
|  |  |  |  |  |  |  |  |  |  |
|  |  |  |  |  |  |  |  |  |  |
| **ClinicalTrials.gov** |  |  |  |  |  |  |  |  |  |
|  | 2015 |  |  |  |  |  |  |  |  |
|  |  |  |  |  |  |  |  |  |  |
|  |  | Mucopolysaccharidosis VII | 1 |  |  | Pharmaceutical Industry | Private | International | 12 |
|  |  |  |  |  |  |  |  |  |  |
|  |  |  |  |  |  |  |  |  |  |
|  | 2020 |  |  |  |  |  |  |  |  |
|  |  | GM2 Gangliosidosis |  | 1 |  | Pharmaceutical industry | Private | International | 74 |
|  |  |  |  |  |  |  |  |  |  |

Source: Prepared by the authors based on data obtained from the digital repositories ReBEC, Plataforma Brasil e ClinicalTrials.gov.

*NR = Not Reported
